# Metabolic characteristics revealing cell differentiation of nasopharyngeal carcinoma by combining NMR spectroscopy with Raman spectroscopy

**DOI:** 10.1186/s12935-019-0759-4

**Published:** 2019-02-18

**Authors:** Yang Chen, Zhong Chen, Ying Su, Donghong Lin, Min Chen, Shangyuan Feng, Changyan Zou

**Affiliations:** 1 Department of Laboratory Medicine, Fujian Medical University, Fuzhou, 350004 China; 2 Department of Electronic Science, Fujian Provincial Key Laboratory of Plasma and Magnetic Resonance, Xiamen University, Xiamen, 361005 China; 3 Laboratory of Radiobiology, Fujian Provincial Tumor Hospital, Fuzhou, 350014 China; 4 Key Laboratory of Optoelectronic Science and Technology for Medicine, Ministry of Education, Fujian Normal University, Fuzhou, 350007 China

**Keywords:** Nasopharyngeal carcinoma, Cell differentiation, Metabolomics, Proton NMR spectroscopy, Raman spectroscopy

## Abstract

**Background:**

The staging system of nasopharyngeal carcinoma (NPC) has close relationship with the degree of cell differentiation, but most NPC patients remain undiagnosed until advanced phases. Novel metabolic markers need to be characterized to support diagnose at an early stage.

**Methods:**

Metabolic characteristics of nasopharyngeal normal cell NP69 and two types of NPC cells, including CNE1 and CNE2 associated with high and low differentiation degrees were studied by combining ^1^H NMR spectroscopy with Raman spectroscopy. Statistical methods were also utilized to determine potential characteristic metabolites for monitoring differentiation progression.

**Results:**

Metabolic profiles of NPC cells were significantly different according to differentiation degrees. Various characteristic metabolites responsible for different differentiated NPC cells were identified, and then disordered metabolic pathways were combed according to these metabolites. We found disordered pathways mainly included amino acids metabolisms like essential amino acids metabolisms, as well as altered lipid metabolism and TCA cycle, and abnormal energy metabolism. Thus our results provide evidence about close relationship between differentiation degrees of NPC cells and the levels of intracellular metabolites. Moreover, Raman spectrum analysis also provided complementary and confirmatory information about intracellular components in single living cells. Eight pathways were verified to that in NMR analysis, including amino acids metabolisms, inositol phosphate metabolism, and purine metabolism.

**Conclusions:**

Methodology of NMR-based metabolomics combining with Raman spectroscopy could be powerful and straightforward to reveal cell differentiation development and meanwhile lay the basis for experimental and clinical practice to monitor disease progression and therapeutic evaluation.

**Electronic supplementary material:**

The online version of this article (10.1186/s12935-019-0759-4) contains supplementary material, which is available to authorized users.

## Background

Nasopharyngeal carcinoma (NPC) is a non-lymphomatous, squamous cell carcinoma that arises from epithelial cells on the surface of nasopharynx. It is a common head and neck cancer in South China and Southeast Asia, where the annual incidence ranges from 20 to 50 per 100,000 people [1]. According to the WHO staging system, the three histologic subtypes, including keratinizing squamous cell carcinoma (type I), nonkeratinizing differentiated carcinoma (type II), and nonkeratinizing undifferentiated carcinoma (type III), are closely related to the degree of cell differentiation. Nonkeratinizing undifferentiated carcinoma comprises the majority of NPC cases in endemic regions such as South China [2]. Due to deep location and vague symptoms, most NPC patients remain undiagnosed until advanced phases with cervical lymph nodes and distant metastasis [3, 4]. Therefore, there is an obvious clinical demand for novel markers from cellular level that help to diagnose at an early stage, especially from metabolic markers associated with cell differentiation. However, current diagnostic method is based on clinical history, endoscopic examination, and a biopsy of the lesion [5], as well as a series of radiological tests [6]. These examinations are not only difficult for early diagnosis of NPC, but also invasive for patients. To improve the overall prognosis and reduce mortality rate of NPC patients, it is critical for researchers to develop effective strategies from cellular level for monitoring differentiation progression and therapeutic evaluation.

Metabolomics platform allows analyses of cell metabolism that is proximal to phenotype and all metabolites of low molecular weight inside cells with given set of physiological activity. Numerous analytical tools have been applied to this endeavor [7, 8], and among them NMR spectroscopy has been playing a significant role in this field. During the last decade, NMR-based metabolomics technology has been successfully used to investigate nutritional, toxicological and proliferation effects in living cell models. Response of bioreactor scale to express recombinant protein and pharmacological effects were investigated in various tumor cells [9]. By NMR-based metabolic profiling, cellular metabolism in a proliferation-arrested cell line was shown to increase productivity via which revealed important metabolic pathways for further detailed studies [10]. Variants inside cell lines with different levels of resistance or different lung tumor origins were also presented to generally demonstrate from both whole cells and extracts [11]. It is interesting that cellular metabolic behaviors and patterns are so complex that are very sensitive to surrounding environment. Dewar et al. [12] demonstrated that chronic myelogenous leukemic cell line and its imatinib resistant subline were quite different in metabolic profiles with an elevation of creatine metabolites in the latter. Progress in understanding metabolic differences of cells related to oncogenic transformation and metastatic potential have also been supported by a few researches. Vered et al. [13] demonstrated that RAS-driven physiologic alterations will affect water soluble metabolites and help to distinguish lung epithelial cells with different RAS oncogenic isoforms. More recent progress was conducted by malignancy-associated cancer cell lines from human and murine models. Metabolic heterogeneity of different degrees of astrocytoma cell lines from glioma tissues was revealed to be associated with malignancy [14]. In addition to discussing the mechanisms for proliferation or resistance, other researchers have turned their attention to cell differentiation arising from process of cell growth. Process of cells differentiation into enterocyte has been investigated to learn more detailed mechanisms, so that levels of certain metabolites were shown to change dramatically between undifferentiated state and late differentiated states [15]. In the case of NPC, only a few studies focus on metabolic profiling of sera samples. For example, Yi et al. demonstrated that gas chromatography-mass spectrometry-based metabolomics analysis could identify stages of NPC subjects via metabolic profiling, and possible therapeutic outcome were evaluated through certain metabolites alterations [16, 17]. Wang et al. [18] also revealed metabolic abnormalities during NPC serum study by NMR spectroscopy. However, neither the underlying metabolism that is characteristic of NPC cell differentiation has not been deeply investigated nor the relationship between differentiation degree and metabolism in cells has not been fully clarified.

In this work, metabolic characteristics of NPC cells associated with different differentiation degrees were performed by ^1^H NMR spectroscopy. Then Raman spectroscopy, an optical technique based on molecular-specific, inelastic scattering of light photons, was also used to verify the extent of screened metabolites and link these metabolites to possible spectral characteristics that were complementary or confirmatory to that of NMR. We aimed to preliminarily understand the molecular mechanisms and determine characteristic metabolites for cell differentiation. To the best of our knowledge, this is the first attempt to explore metabolic profiles of NPC cells by combining NMR-based metabolomics and Raman spectroscopy.

## Methods

### Cell lines for construction of differentiation model

Two cell lines were chosen to represent the differentiation of neoplastic development in the nasopharynx, including human NPC cell lines CNE1 (high differentiated) and CNE2 (low differentiated). The CNE1 and CNE2 were the first and second strains established by the Chinese Academy of Preventive Medicine and have been widely used in various researches. The differentiation characteristics have also been confirmed by other report [19]. In this study, these cell lines were commercially purchased from the company (Taisheng Biotech., Guangzhou, China) and cultured in RPMI-1640 medium supplemented with 100 U/mL streptomycin, 100 U/mL penicillin, and 10% Newborn Calf Serum (Invitrogen Corporation, USA). Another nasopharyngeal normal NP69 (Xiangya Central Experiment Laboratory, Changsha, China) cultured in Keratinocyte-SFM medium (Invitrogen Corporation, USA) was chosen to represent mature cell line. All cells were grown in the optimal media under an incubator humidified 5% CO_2_ atmosphere at 37 °C. Fresh stock of cells was seeded in 10 identical flasks until the cells entered into logarithmic growth phase. Then these flasks were divided into two groups for NMR and Raman measurement, respectively (i.e. six flasks for NMR; four for Raman).

### Preparation of cellular metabolites and NMR experimentation

Cultured media was separately collected before cell harvesting. Cells with quantity of approximately 1 × 10^6^/mL in every flask were harvest. Through a dual phase extraction procedure, intracellular metabolites were extracted by mixture of methanol, chloroform and water under volume ratio of 2:2:3 [13, 14]. Aqueous-phase extracts were then dissolved in 500 μL of D_2_O-prepared phosphate-buffered saline (PBS, pH = 7.4). For media samples, 400 μL of the media was mixed with 100 μL of D_2_O-prepared PBS (pH = 7.4) containing 0.05% sodium 3-(trimethylsilyl) propionate-2,2,3,3-d_4_ (TSP) which was served as reference (δ0.00). Before NMR experiment, both media and cell samples were oscillated for blending and centrifuged at 10,000 rpm for 10 min at 4 °C to remove insoluble components. The supernatant (500 μL) was transferred to 5 mm NMR tube for later measurement. Finally, due to failure preparation, one to three samples lost detectability for cell or cultured media.

The NMR measurements were performed at 298 K on a Bruker Avance III 600 MHz spectrometer, operating at a ^1^H frequency of 600.13 MHz. Solvent suppressed 1D NOESY spectra were acquired using a pulse sequence [relaxation delay-90º-t_1_-90º-t_m_-90º-acquisition] with acquisition time of 2 s. And low-power water signal presaturation was also used during both the 4 s relaxation delay and the 120 ms mixing time (t_m_). The spectral width was 12 kHz with an acquisition time per scan of 2.65 s, and 256 transients were collected into 32 K data points for each spectrum.

### Cell preparation and Raman spectra acquisition

Cells in logarithmic growth phase were made into suspension in media to maintain activity prior to Raman measurement. Then the suspension was deposited on a rectangle-aluminum plate. Raman spectra of single living cells were recorded using a Renishaw InVia Micro-Raman system with a 50 × objective with a numerical aperture of 0.75. Laser beam from a 785 nm multimode high power diode (about 20 mW of power) was used for excitation. The diameter of focal area for collecting spectra was approximately 5 μm which was smaller than a cell, thus spectra were collected at three points of a triangle inside the cell, resulting in full coverage of focal area on the cells. The spectra were recorded with 20 s of integration time in the range of 300 to 1800 cm^−1^. All measurements were finished in a few minutes and the sample was subsequently replaced by a new one to restart Raman measurement. Finally, three spectra of a single cell were averaged to obtain mean spectra which would be gathered according to corresponding cell types, resulting in spectral quantity of 30 for every cell line.

### Data preprocessing and statistical analysis

For NMR data, all free induction decays were multiplied by a 0.3 Hz line-broadening factor prior to Fourier transformation. Then all NMR spectra were manually phased and baseline-corrected via MestReNova (version 10.0.0, Mestrelab Research S.L., Spain). The spectra of media and cell extracts were referenced to signals of TSP (δ0.00) and lactate methyl (δ1.33), respectively. The region of δ4.75–5.00 ppm was excluded to remove effects of residual water variation. After that, the spectra were divided into 0.002 ppm integral regions and integrated in the region of 0.5–9.5 ppm (for cell extracts) or 0.5–9.0 ppm (for cultured media). The spectra were subsequently normalized to the total sum of the spectrum, which compensate for variations in sample concentration.

Principal components analysis (PCA) and orthogonal projection to latent structure with discriminant analysis (OPLS-DA) were carried out using SIMCA-P + (version 14.0, Umetrics, Sweden). Optimal number of orthogonal components for building OPLS-DA model was selected using cross validation procedure and the goodness of fit and prediction parameters (R^2^ and Q^2^) were calculated. For screening discriminatory metabolites, cut-off value of correlation coefficient was determined by degree of freedom in the OPLS-DA model. Those discriminatory metabolites with significance level of *P *< 0.05 according to Student’s *t* test analysis were included in the final list of characteristic metabolites. Based on characteristic metabolites, a MATLAB-based toolbox was used to draw the map of relative biochemical pathways [20], and custom sub-networks were created by using main substrate-product pairs as defined by Kyoto encyclopedia of genes and genomes (KEGG) online database.

For Raman data, all mean spectra of single cells were extracted by background auto-fluorescence subtraction using Vancouver Raman Algorithm as demonstrated by Zhao et al. [21], and then averaged. We further normalized these mean spectra according to the area under the curve so as to eliminate the effect of the system.

## Results

### Metabolic profiles of nasopharyngeal carcinoma cells differed from differentiation

High quality of ^1^H NMR spectra from cell and media samples (Additional file 1: Figure S1), including control media are acquired. Individual metabolites are further assigned (see Additional file 1: Figure S2 and Table S1) according to the literature data and confirmed by Human Metabolome Database (http://www.hmdb.ca) [22–26]. Various signals were assigned to individual metabolites and provided adequate information to assess variations in metabolic profiles within those cells. In the ^1^H NMR spectra, aliphatic regions are dominated by various metabolites, containing numerous resonances from amino acids like essential amino acids (EAAs, including isoleucine, leucine, valine, lysine), non-essential amino acids (alanine, methionine, glycine, and glutamate), TCA intermediates (lactate and succinate), and others metabolites. The low field region represents chemical shifts of the aromatic nucleoside (tyrosine and phenylalanine) and ribose signals (ADP, ATP) as well as metabolic waste. Inspection the spectra of cell extract revealed some obvious metabolic differences among these cell lines, and that differences in some metabolites concentrations were linked to major alterations in metabolisms which occur in tumorigenic cells (Additional file 1: Figure S1A–C). Moreover, the NMR spectra of cultured media were characterized by various necessary nutritional components including amino acids and glucose to support cellular growth (Additional file 1: Figure S1D–F). Since compositional changes in cultured media reflected not only consumption of nutrients but also the physiological function of cells, metabolic end-products and intermediates, such as the intermediates of glycolysis, TCA (pyruvate, acetate, and succinate) as well as metabolic waste were observed. However, to get more detailed metabolic variations between normal and NPC cells and between high and low differentiated NPC cells, more precise information need to be confirmed by further multivariate analysis so as to determine characteristic differences.

### Characteristic metabolites associated with high and low differentiated cells

**Fig. 1:**
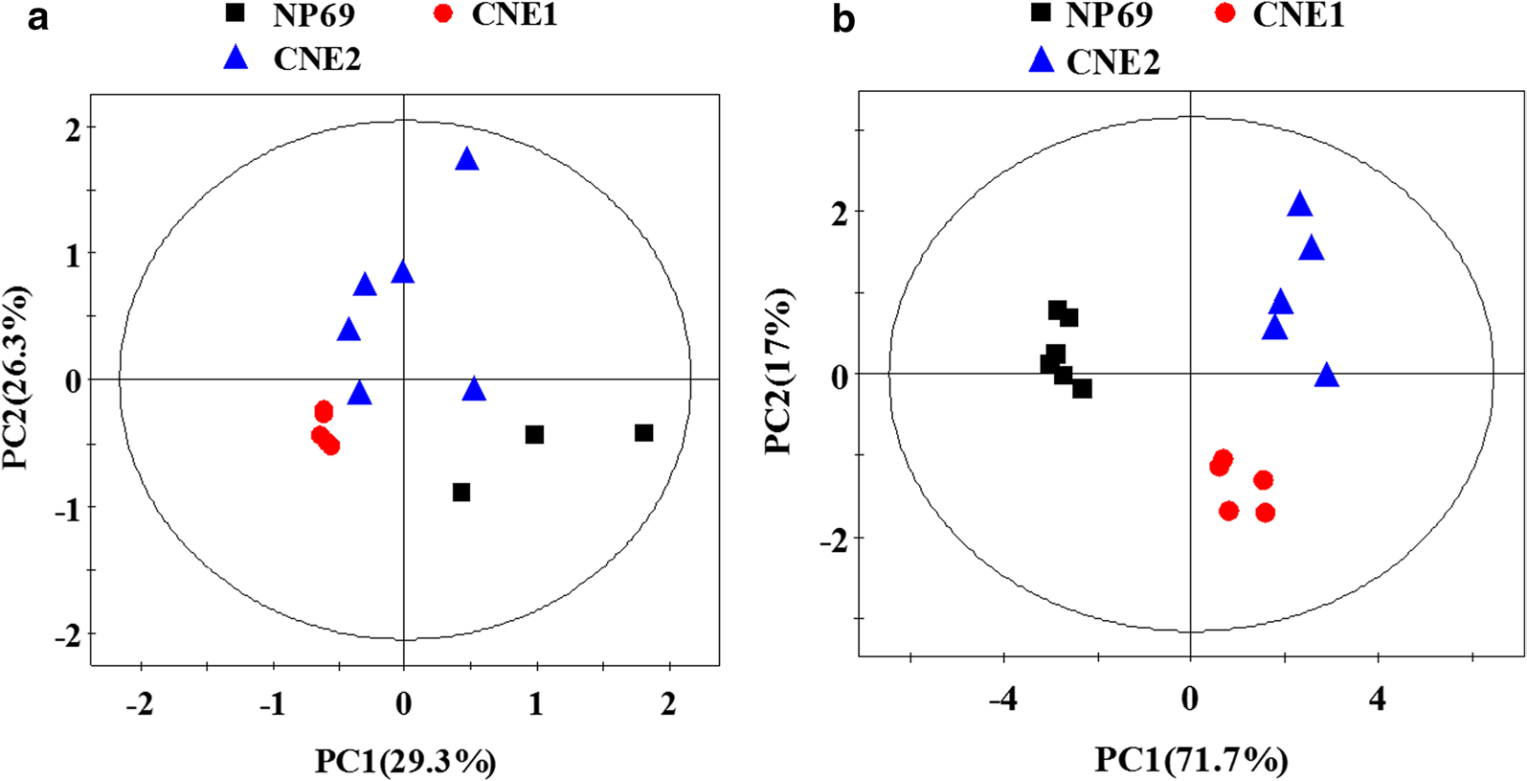
Scores plots in PCA analysis for NMR spectral data from cell extracts and cultured media. **a** Cell extracts, **b** cultured media

We firstly performed PCA on the normalized ^1^H NMR spectra from both cell extracts (Fig. 1a) and cultured media (Fig. 1b). Class separation in both models is reasonably good, particularly considering that this is an unsupervised model of three classes, each of which contains only three to six members. In fact, performance of the media model may be, in some ways, preferable to that of cell extracts for this data set. For example, 71.7% of the variation in the media data was captured in PC1, whereas only 29.3% was captured for cell extracts data. Some information from the PCA models was consistent, but some appeared to be complementary. For both models, cell line from normal nasopharynx was clearly separated from those derived from high- and low-differentiated NPC (CNE1 and CNE2) along the principal component.

**Fig. 2:**
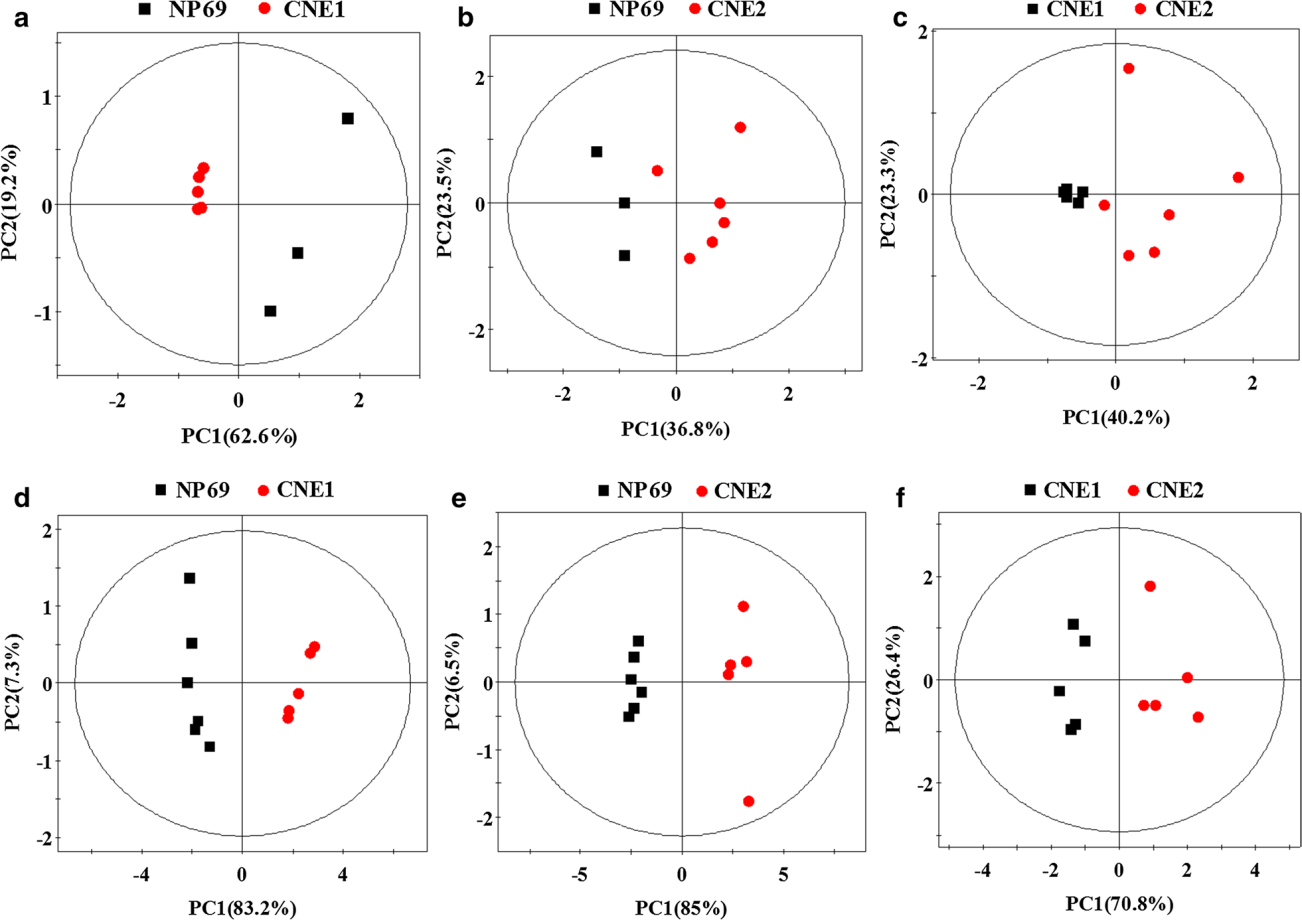
Scores plots in pairwise PCA analysis from cell extracts (**a**–**c**) and cultured media (**d**–**f**)

Also, note that the PCA scores plot exhibited different trends and dispersion which were confirmed by pairwise comparisons, as shown in Fig. 2. Interestingly, in most case of this figure, if we consider NP69 as mature cell, tumor cells closer to mature cells were mostly located on the left side of the principal component (except cell extracts model from NP69 vs. CNE1), indicating that PCA analysis reflected not only the statistical differences among classes, but also the relationship between cell differentiation and maturity. The plots demonstrated that both cell extracts and cultured media of normal and different differentiated NPC cell lines presented different metabolic profiles due to inherent biological character.

**Fig. 3:**
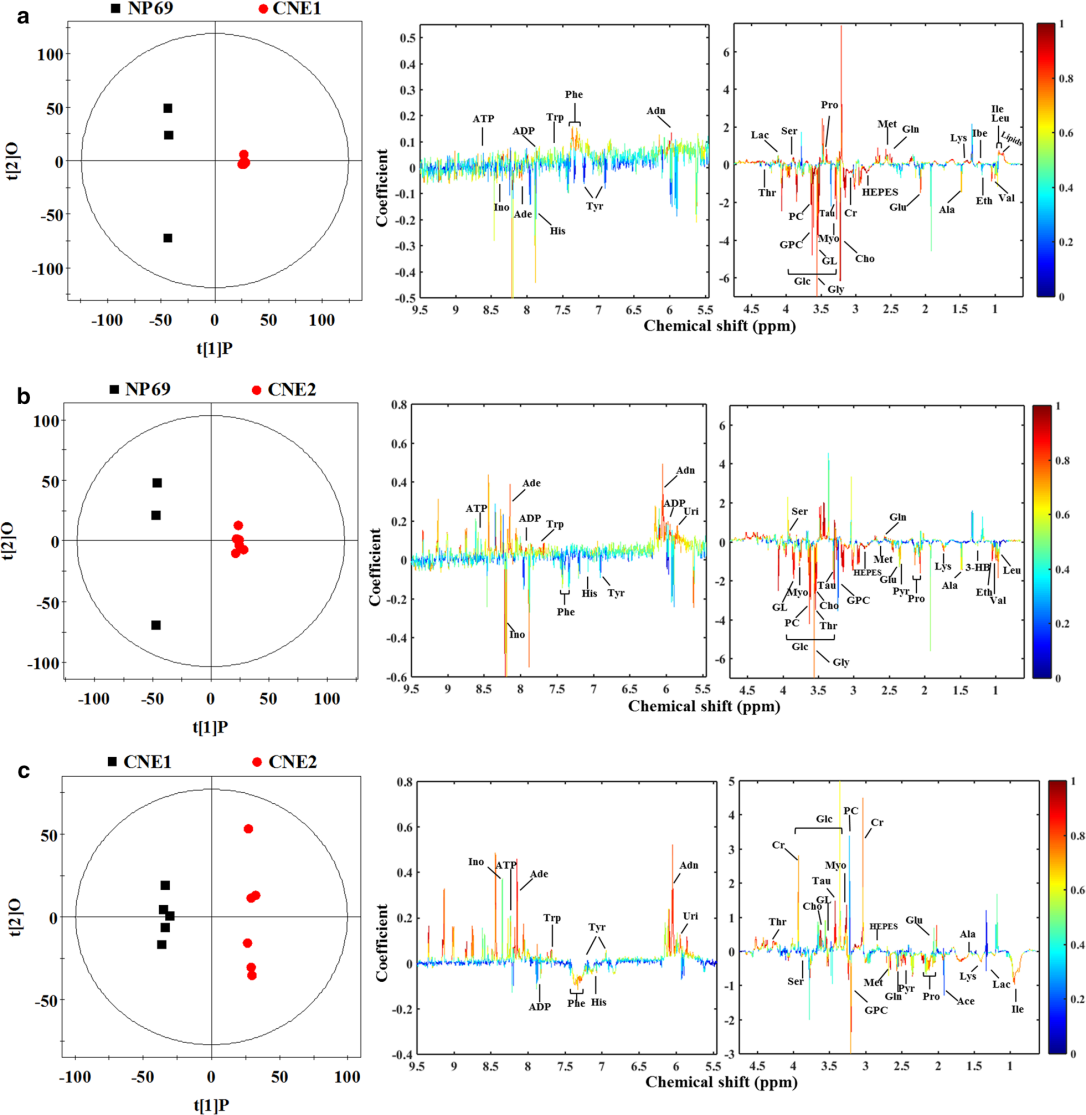
Scores and loading plots in pairwise OPLS-DA analysis from cell extracts. **a** Normal NP69 versus high differentiated CNE1 cells (R^2^X = 81.6%, R^2^Y = 0.999, Q^2^ = 0.775, *P *< 0.0001), **b** normal NP69 versus low differentiated CNE2 cells (R^2^X = 77.8%, R^2^Y = 0.997, Q^2^ = 0.827, *P *< 0.0001), **c** high differentiated CNE1 versus low differentiated CNE2 cells (R^2^X = 57.6%, R^2^Y = 0.996, Q^2^ = 0.713, *P *< 0.0001)

**Fig. 4:**
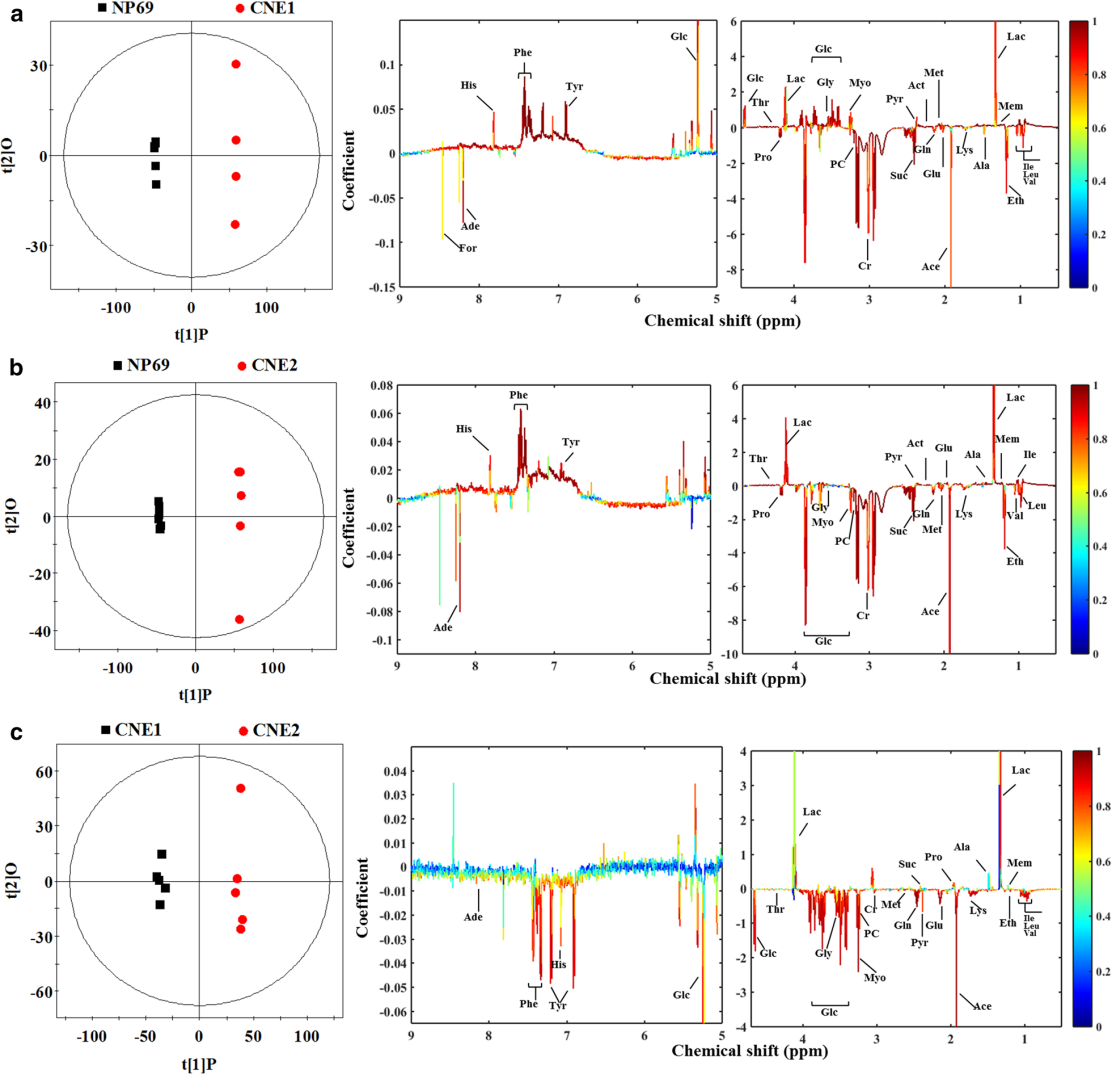
Scores and loading plots in pairwise OPLS-DA analysis from cultured media. **a** Normal NP69 versus high differentiated CNE1 cells (R^2^X = 83.6%, R^2^Y = 1.000, Q^2^ = 0.995, *P *< 0.0001), **b** normal NP69 versus low differentiated CNE2 cells (R^2^X = 81.7%, R^2^Y = 1.000, Q^2^ = 0.976, *P *< 0.0001), **c** high differentiated CNE1 versus low differentiated CNE2 cells (R^2^X = 67.0%, R^2^Y = 0.996, Q^2^ = 0.773, *P *< 0.0001)

In addition to modeling normal and different differentiated NPC cell lines simultaneously to identify characteristic metabolites responsible for the separation, and meanwhile determining how well these metabolites correlate with the differentiation degrees, we applied OPLS-DA to analyze the metabolic profiles in a pairwise fashion. The scores and loading plots from those pairwise models were used to determine some of the metabolites that were responsible for class separation. As a result, those significantly discriminatory metabolites were identified from cell extracts (Fig. 3) and cultured media (Fig. 4). Based on this, relative concentrations were compared for further determination of characteristic metabolites responsible for the clustering patterns. Metabolites considered significant (P < 0.05) were included in the final list of characteristic metabolites which were considered to be responsible for alterations due to different differentiation degrees.

**Fig. 5:**
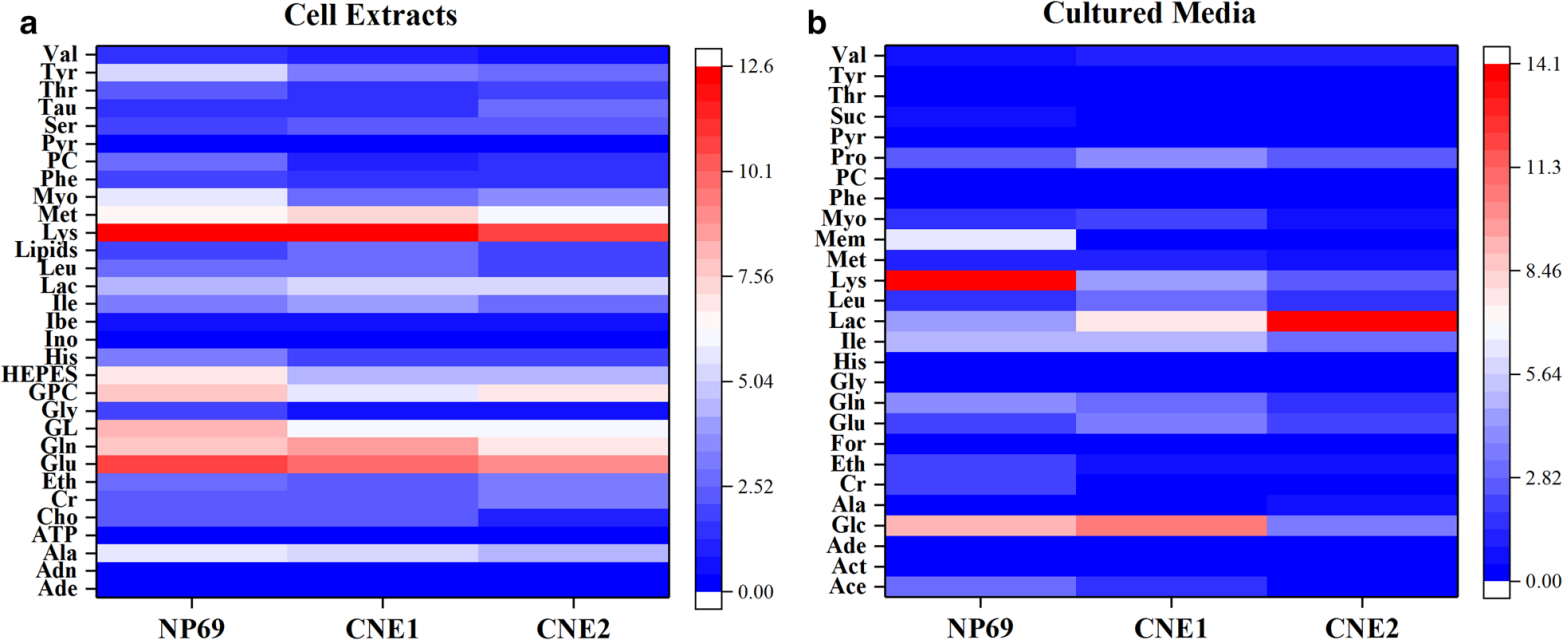
The heat map of characteristic metabolites contents in the cell extracts (**a**) and cultured media (**b**)

According to list of characteristic metabolites from cell extracts (Table 1) and the visualization from heat map of metabolite contents (Fig. 5a), comparing with normal cell, levels of most characteristic metabolites decreased and the majority was made up of amino acids. Only four metabolites were elevated in each NPC cell, and these metabolites included only one amino acid. Moreover, there were eleven common characteristic metabolites for both sides, more than half of which came from amino acids, and interestingly, levels of all these common characteristic metabolites were lower than normal cell except taurine. Since the level of taurine decreased in CNE1, whereas increased in CNE2, indicating not only some alterations in amino acid metabolisms from mature cell aspect, but also distinct metabolic profiles from differentiation characteristics. During comparison of high and low differentiated NPC cells, eighteen characteristic metabolites were screened (half increased and the other half decreased), and five of them were similar with those above-mentioned common characteristic metabolites from normal cell. Notably, eight amino acids, including alanine, glutamate, glutamine, isoleucine, lysine, methionine taurine, and threonine, were observed with significant changes between high and low differentiated NPC cells. Only taurine and threonine were elevated in low differentiated CNE2 cell, while the others decreased, including three EAAs (isoleucine, lysine, and methionine).

**Table 1:** Concentrations of characteristic metabolites derived from cell extracts

Metabolite	NP69	CNE1	CNE2
Adenine	0.06 ± 0.02^a^	0.04 ± 0.00	0.10 ± 0.02^b,c^
Adenosine	0.12 ± 0.06	0.05 ± 0.01	0.21 ± 0.05^b,c^
Alanine	5.61 ± 0.53	5.25 ± 0.07	4.58 ± 0.12^b,c^
ATP	0.09 ± 0.08	0.14 ± 0.03	0.33 ± 0.05^b,c^
Choline	2.51 ± 0.30	2.47 ± 0.06	1.18 ± 0.15^b,c^
Creatine	2.34 ± 0.29	2.15 ± 0.01	3.32 ± 0.11^c^
Ethanol	2.79 ± 0.18	2.21 ± 0.10^b^	3.12 ± 0.48
Glutamate	10.76 ± 0.99	9.77 ± 0.10	9.02 ± 0.27^c^
Glutamine	7.63 ± 0.97	8.45 ± 0.10	7.00 ± 0.26^c^
Glycerol	8.22 ± 1.11	6.18 ± 0.05^b^	5.90 ± 0.32^b^
Glycine	1.72 ± 0.19	0.44 ± 0.02^b^	0.60 ± 0.05^b^
GPC	7.62 ± 0.66	5.68 ± 0.04^b^	6.84 ± 0.73
HEPES	7.14 ± 0.80	4.54 ± 0.03^b^	4.24 ± 0.12^b,c^
Histidine	3.31 ± 0.19	1.82 ± 0.03^b^	1.97 ± 0.07^b^
Inosine	0.27 ± 0.07	0.18 ± 0.01	0.27 ± 0.03^c^
Isobutyrate	0.52 ± 0.04	0.63 ± 0.01^b^	0.54 ± 0.03
Isoleucine	3.22 ± 0.49	3.89 ± 0.08	2.93 ± 0.24^c^
Lactate	4.28 ± 0.32	5.21 ± 0.16^b^	5.27 ± 0.77
Leucine	2.67 ± 0.27	2.54 ± 0.09	1.93 ± 0.16^b^
Lipids	1.84 ± 0.42	2.80 ± 0.05^b^	2.03 ± 0.22
Lysine	12.18 ± 0.40	12.60 ± 0.15	10.87 ± 0.64^c^
Methionine	6.69 ± 0.56	7.36 ± 0.11	6.13 ± 0.12^c^
*Myo*-inositol	5.87 ± 0.63	2.77 ± 0.03^b^	3.65 ± 0.24^b,c^
Phenylalanine	1.82 ± 0.11	1.44 ± 0.04^b^	1.40 ± 0.07^b^
Phosphorylcholine	2.68 ± 0.27	1.12 ± 0.01^b^	1.30 ± 0.05^b,c^
Pyruvate	0.18 ± 0.02	0.17 ± 0.02	0.12 ± 0.01^b,c^
Serine	2.06 ± 0.14	2.35 ± 0.04^b^	2.25 ± 0.09
Taurine	1.58 ± 0.06	1.36 ± 0.05^b^	2.52 ± 0.12^b,c^
Threonine	2.51 ± 0.27	1.40 ± 0.03^b^	1.71 ± 0.08^b,c^
Tyrosine	5.11 ± 0.33	2.97 ± 0.05^b^	2.80 ± 0.11^b^
Valine	1.33 ± 0.17	0.98 ± 0.04^b^	0.82 ± 0.06^b^

^a^The concentrations of metabolites are presented as mean ± SE of the integration value of the characteristic resonance of each metabolite

Characteristic metabolite with *P *< 0.05 versus: ^b^ normal NP69

^c^High differentiated CNE1

Under the same screening strategy, media-based characteristic metabolites were also filtered out (see Additional file 1: Table S2) and visualized from heat map (Fig. 5b). Compared with normal cell (NP69), only a few metabolites did not show to change. High and low differentiated NPC cells shared seventeen common characteristic metabolites, accounting for more than 85% of the characteristic metabolites. These common metabolites have the same up- or down-regulation in NPC cells only except *myo*-inositol, because its level goes up in high differentiated CNE1 cell but down in low differentiated CNE2 cell. Besides, nineteen characteristic metabolites were screened when comparing high and low differentiated NPC cells, in which all of them decreased in low differentiated cell with the exception of an increase in lactate. It was also found that eleven of these characteristic metabolites were similar with those above-mentioned common characteristic metabolites when compared with normal cell.

### Metabolic pathway analysis revealing altered differentiation processes

**Fig. 6:**
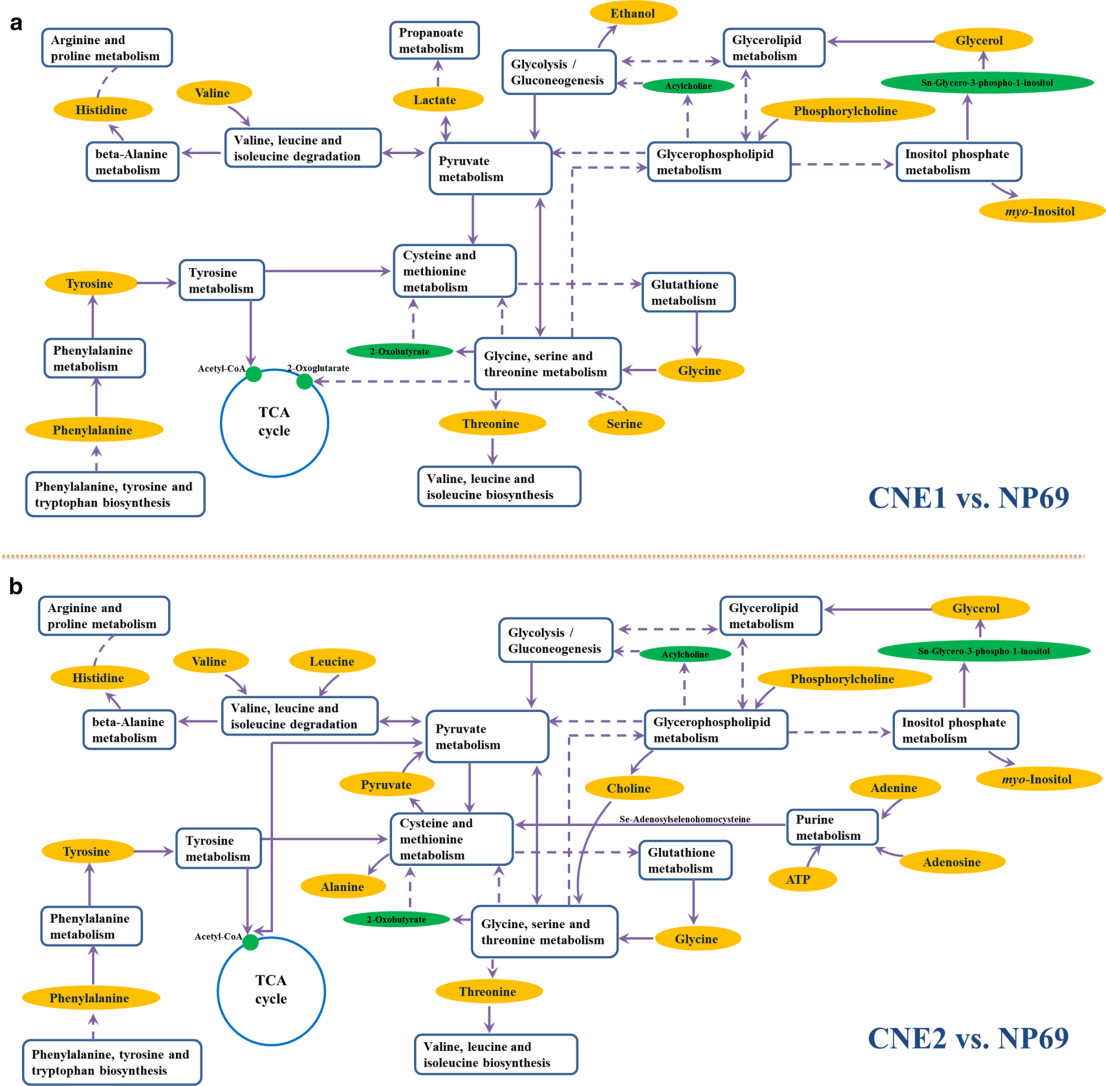
Schematic diagram of metabolic pathways associated with NPC cell differentiation development. The metabolites in orange backgrounds were characteristic metabolites with significant changes from cell extracts when compared with normal cells, and the corresponding disordered pathways were demonstrated for high differentiated CNE1 (**a**) and low differentiated CNE2 (**b**), respectively

With the help of KEGG online database, the potential disordered metabolic networks of NPC cell lines associated with cellular differentiation behaviors could be rationally derived. As shown in Fig. 6, based on characteristic metabolites from cell extracts, the common pathways determined to under-concentrate in NPC cell differentiation are TCA cycle, energy metabolism (i.e., glycolysis, glycerolipid metabolism, glycerophospholipid metabolism), as well as several amino acid metabolisms. Seven kinds of EAAs were involved in the behaviors of metabolisms or degradation and biosynthesis. The branched-chain amino acids (BCAAs, i.e., isoleucine, leucine, and valine) were observed and together with phenylalanine and tyrosine to contribute main amino acids biosynthesis or degradation. It seems that more specific pathways are involved for CNE2 cell, suggesting more serious disorders in low differentiated cell. One of the BCAAs, the level of leucine decreased significantly to around 70% of normal level, revealing some alterations in degradation of BCAAs.

### Raman spectral characterization of single cell and metabolic profile evaluation

**Fig. 7:**
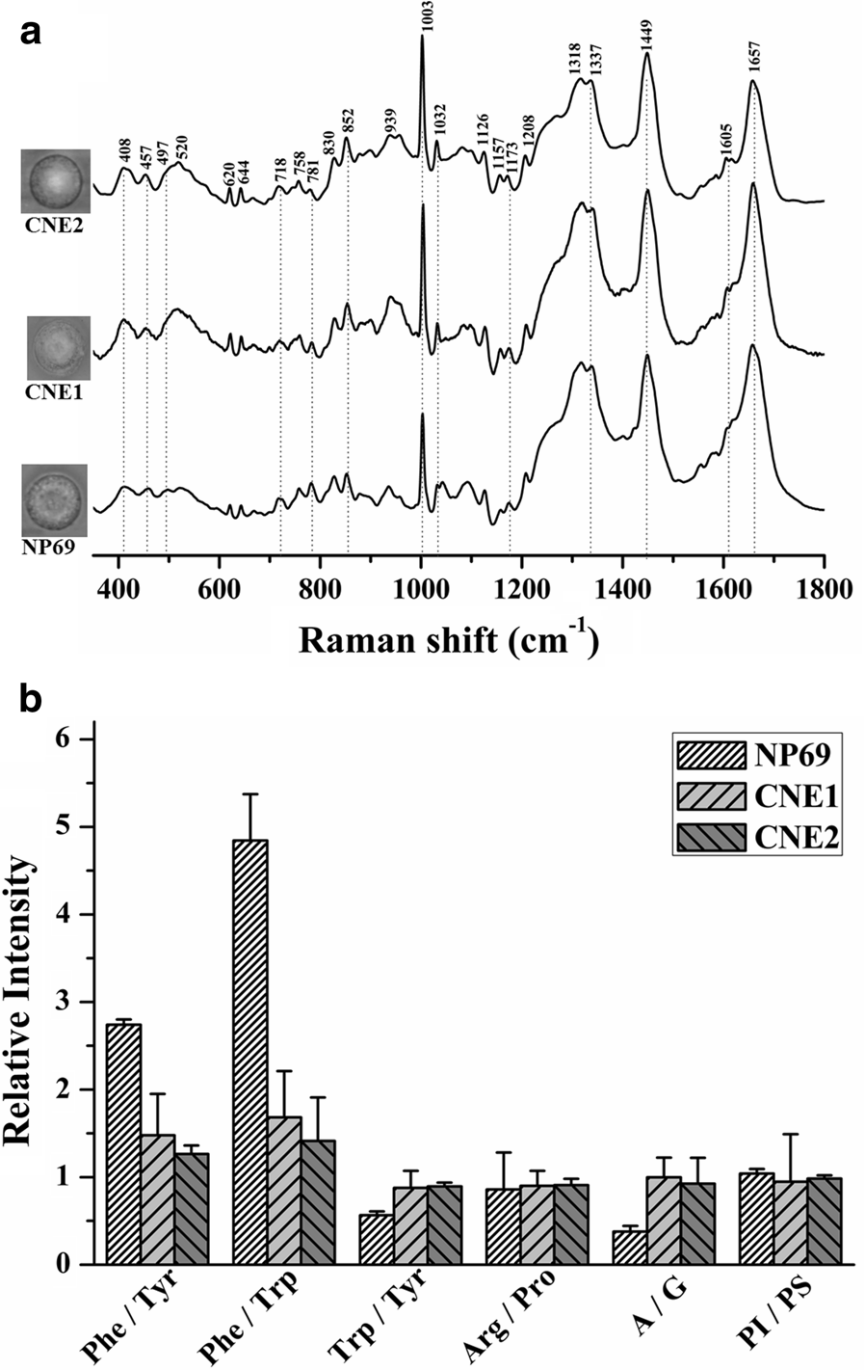
Comparison of mean Raman spectra and relative contents of certain metabolites in single living cells. **a** Mean Raman spectra, **b** relative intensity of metabolites within cells

Here, we also report on Raman spectroscopy to detect intracellular components in single living cells. Like NMR, the Raman approach also provides a rapid “snapshot” of the vibration mode from aqueous samples and may be possible to obtain information from molecular level that is complementary or confirmatory to that of NMR. Mean Raman spectra with good quality were collected from those two types of NPC cells and nasopharyngeal normal cell. As shown in Fig. 7a, it is difficult to see whether differences exist among these spectra, because these spectra exhibit almost similar spectral profiles, indicating same components in cells. Several prominent Raman bands can be consistently observed in both normal and cancer cells and then assigned according to previous work (see Additional file 1: Table S3) [27–30].

Some Raman bands were identified as metabolites that either already existed in NMR spectra, or only found in Raman spectra. These metabolites were summarized in Table 2, together with the determination of biological function by combining the above metabolic pathway analysis and KEGG database. As a result, eight pathways were involved, including several amino acid metabolisms, inositol phosphate metabolism, and purine metabolism. According to the biological function associated with different kinds of metabolic pathways, several ratios of Raman band intensity were compared between normal NP69 cell and NPC cells in order to explore whether relative pathways have become disordered. As shown in Fig. 7b, pairwise ratios of three amino acids (phenylalanine, tyrosine, and tryptophan that specifically located at 1003, 852, and 457 cm^−1^ respectively) seem to change dramatically from normal NP69 to different differentiated NPC cells. For example, the ratios of phenylalanine to tyrosine and tryptophan in normal NP69 was almost twofold as in NPC cells, and these ratios decreased gradually with differentiation changing to low degree, thus the relative pathway may obviously become disordered. Another significant change came from purine of DNA/RNA, in which adenine (716 cm^−1^) and guanine (1041 cm^−1^) were involved in purine metabolism, and we found that their ratios were 2.4 to 2.6 fold higher in different differentiated NPC cells than in normal cell. We are concerned about two metabolites of phosphoglyceride, including phosphatidylinositol (PI, 408 cm^−1^) and phosphatidylserine (PS, 523 cm^−1^). The ratio of PI to PS was greater than 1.0 in normal cell, but less than 1.0 in NPC cells. This is very interesting and further validates the abnormal lipid metabolisms deduced by the above results of NMR measurement.

**Table 2:** Raman assignments of metabolites from single cells and their biological function

Metabolite	Raman shift (cm^−1^)	Biological function/interpretation
Phosphatidylinositol	408	Inositol phosphate metabolism
Arginine	497	Arginine and proline metabolism
Proline	935	
Phosphatidylserine	523	Glycine, serine and threonine metabolism
Tryptophan	457, 758, 1207	Phenylalanine, tyrosine and tryptophan biosynthesis
Phenylalanine	621, 644, 1003, 1032, 1207, 1605	(1) Phenylalanine, tyrosine and tryptophan biosynthesis (2) Phenylalanine metabolism
Tyrosine	644, 827, 852, 1174, 1605	(1) Phenylalanine, tyrosine and tryptophan biosynthesis (2) Tyrosine metabolism
Valine	935	Valine, leucine and isoleucine degradation
Adenine	716, 1207, 1337	Purine metabolism
Guanine	1041, 1317, 1337	
Thymine	758, 1207	
Uracil	782	
Cytosine	782	

## Discussion

The two cell lines used in this study were derived from NPC tissue of different differentiation of neoplastic development in the nasopharynx. Nasopharyngeal normal cell line NP69 was used as reference to analyze cellular metabolome and metabolites compositional changes in cultured media response to cell differentiation. The NMR results have clearly demonstrated that there were significant differences in amino acid and other metabolites between high and low differentiated cells. Notably, as TCA intermediate, higher production of lactate into media may reflect altered TCA cycle and the utilization of less efficient glycolytic pathway of low differentiated cell. Abnormal energy metabolism in cancer cells as described in Warburg’s theory promotes the synthesis of 2-oxoglutarate, which is the metabolic intermediate of TCA cycle in mitochondria, thereby also facilitating the synthesis of nucleotides required for DNA replication and cellular proliferation in low differentiated cell and subsequently increase accumulation of lactate [31, 32]. Therefore, the metabolic reprogramming in cancer cells, which is orchestrated by the increased expression and the interaction of amino acid transporters, is expected to protect cancer cells from accumulated reactive oxygen species via the robust GSH synthesis [33]. Other proposed characteristic metabolites from cultured media were down-regulated, indicating that the nutrition in media could effectively support the proliferation of low differentiated cell, and therefore cause more active energy metabolism and nutrient consumption, as well as the resulting metabolic products. There is evidence that BCAAs are involved in anaplerosis, protein synthesis or catabolizing into sources for glucose and lipid production during tumor cell proliferation and growth. The resulting products may affect proteolysis and cell cycle progression related to cancer development or cachexia [34]. Our results further confirm their critical role in contribution to progression of cell differentiation and indicate abnormal amino acid metabolisms occur in NPC cells.

The exploration of discriminant pathways derived from cell extracts imply that glycerol and *myo*-inositol were down-regulated and involved in altered energy metabolism like glycerolipid and inositol phosphate metabolisms. We found that high and low differentiated NPC cells have their specific metabolisms. For example, high differentiated CNE1 cell tends to produce very different level of lactate via pyruvate metabolism for further involvement in propanoate metabolism. The ethanol level may reflect alteration of energy consumption and significantly decreased ethanol level suggested more active aerobic glycolysis or the Warburg effect in CNE1 than that of CNE2 [35]. Our inference is consistent with metabolic symbiosis between hypoxic and oxidative cancer cells, that oxidative cells utilize lactate as a substrate of the TCA cycle, and consequently exhibit stem-like characteristics [36, 37]. Other products of cysteine and methionine metabolism like alanine and pyruvate are essential for the synthesis of protein and phospholipids. It is reported that these metabolites appear to precede enhanced glucose uptake and glycolysis during tumorigenesis [38–40]. Our results show that both of these metabolites decreased, so we may suggest an excessive loss of energy become possible way via purine metabolism, where the demand of ATP is typically increasing in low differentiated CNE2 cell, implying some inherent connection between low differentiation development and altered energy consumption. In addition, choline and its metabolite (phosphorylcholine) are components of phospholipid and cell membrane. They play essential roles in several metabolisms of amino acids and lipid metabolism to keep the integrity of membrane, but their levels fell to less than half of normal level. The reason for this phenomenon is still unclear. Other research has shown that choline level in NPC patients is significantly higher than that in normal control so as to maintain the integrity of cell membrane [18, 41]. Interesting, our results from NPC cells don’t fully agree with this view, and the opposite direction seems to be more rational. There is evidence that cancer stem-like cells expressing high levels of a CD44 variant containing exons 8–10 (CD44v8-10) are resistant to oxidative stress due to the robust glutathione synthesis mediated by stabilization of xCT (a cysteine/glutamate antiporter) at the cellular membrane, resulting in accumulation of some pathogenic proteins in cancer stem-like cells and leading to carcinogenesis [42–44]. Since significant reduction in choline level means cell membrane damage, whereas cell membrane damage occurs frequently due to the fact that low differentiated CNE2 cell exhibits more active cell mitosis than both high differentiated CNE1 and normal cells.

The advantages for NMR-based metabolomics to monitor metabolic profile of NPC cells and their disordered biological functions are undeniable because of the excellent selectivity that is afforded [45, 46]. But there is also considerable interest in evaluating the potential for a wider range of analytical tools that may confirm or complement the information from NMR. Combination of Raman spectroscopy and NMR spectroscopy has been used as metabolomics tool for evaluating the impacts of exposure to environmental contaminants [47]. The potential to profile metabolites in body fluids with Raman spectroscopy has been recognized but not yet fully exploited for cell metabolomics studies [48]. The present Raman spectroscopy of single living cells reflect certain biological behaviors associated with NPC pathogenesis, typically including up-regulated nucleic acid content due to massive cell proliferation and the resulting high demand for energy. There is evidence that gene mutation-mediated nuclear export during NPC cell growth may induce low expression of some oncoprotein which will support the dysregulation of purine metabolism [49]. The Raman results further confirmed that phenylalanine metabolism and tyrosine metabolism could be involved in anaplerosis, and together with 2-oxobutyrate from glycine, serine and threonine metabolism for further involvement in cysteine and methionine metabolism [50]. Typically elevated serine may enter TCA cycle through glycine, serine and threonine metabolism and the subsequent intermediate (2-oxoglutarate). So according to these features of amino acid metabolisms, NPC cells are typically accompanied by BCAAs and EAAs metabolisms and may progress to high and low differentiated cell with certain molecular events. In general, the detailed molecular mechanism is remained to be further elucidated. Our preliminary results also suggest imminence requirement to systematically pursue metabolic characteristics in clinical NPC subjects before promoting further application of these potential diagnostic markers.

## Conclusions

In summary, we provide compelling evidence about changes due to NPC cell differentiation degrees and abnormal metabolic behaviors. Characteristic metabolites responsible for different differentiated NPC cells and relative disordered metabolic pathways were combed, involving EAAs metabolisms, altered lipid metabolism and TCA cycle, as well as abnormal energy metabolism. Further Raman spectral results highlight the molecular information about intracellular components in the level of single living cells and again verify the metabolic profiles to that in NMR. Our results suggested that combination of NMR and Raman spectroscopy could be a potential method for understanding the mechanisms of NPC cell differentiation.

## Additional file

**Additional file 1: Figure S1.** All individual NMR spectra of cell and media samples. **Figure S2.** Representative 600 MHz ^1^H NMR spectra of cell extracts and cultured media. **Table S1.** Assignments of metabolites from ^1^H NMR spectra of NPC cell extracts and cultured media. **Table S2.** Concentrations of characteristic metabolites derived from cultured media. **Table S3.** Main Raman band positions and the corresponding assignments of single living cells.

## Abbreviations

Ace: acetate
Act: acetone
Ade: adenine
Ala: alanine
BCAA: branched-chain amino acids
Cho: choline
Cr: creatine
EAAs: essential amino acids
Eth: ethanol
FMA: fumarate
For: formate
Glc: α-glucose & β-glucose
Glu: glutamate
Gln: glutamine
GL: glycerol
GPC: glycerophosphocholine
Gly: glycine
His: histidine
3-HB: 3-hydroxybutyrate
Ino: inosine
KEGG: Kyoto encyclopedia of genes and genomes
Myo: *myo*-inositol
Ibe: isobutyrate
Ile: isoleucine
Lac: lactate
Leu: leucine
Lys: lysine
Mal: maleate
Met: methionine
Mem: methylmalonate
NPC: nasopharyngeal carcinoma
NMR: nuclear magnetic resonance
OPLS-DA: orthogonal projection to latent structure with discriminant analysis
Phe: phenylalanine
PBS: phosphate-buffered saline
PI: phosphatidylinositol
PS: phosphatidylserine
PC: phosphorylcholine
PCA: principal components analysis
Pro: proline
Pyr: pyruvate
Ser: serine
Suc: succinate
Tau: taurine
Thr: threonine
TCA: tricarboxylic acid
TSP: 3-(trimethylsilyl) propionate-2,2,3,3-d_4_
Trp: tryptophan
Tyr: tyrosine
Uri: uridine
Val: valine
