# Supplementary material for: Metabolic characteristics revealing cell differentiation of nasopharyngeal carcinoma by combining NMR spectroscopy with Raman spectroscopy
Source: Cancer Cell Int. 2019 Feb 18;19:37. doi: 10.1186/s12935-019-0759-4 (PMC6378732; doi:10.1186/s12935-019-0759-4)
Supplement: Supplementary file 1 — Additional file 1: Figure S1. All individual NMR spectra of cell and media samples. Figure S2. Representative 600 MHz 1H NMR spectra of cell extracts and cultured media. Table S1. Assignments of metabolites from 1H NMR spectra of NPC cell extracts and cultured media. Table S2. Concentrations of characteristic metabolites derived from cultured media. Table S3. Main Raman band positions and the corresponding assignments of single living cells. [file 12935_2019_759_MOESM1_ESM.docx]

Metabolic characteristics revealing cell differentiation of nasopharyngeal carcinoma by combining NMR spectroscopy with Raman spectroscopy

Yang Chen^1,2*^, Zhong Chen^2^, Ying Su^3^, Donghong Lin^1*^, Min Chen^1^, Shangyuan Feng^4^ and Changyan Zou^3^

Address: ^1^ Department of Laboratory Medicine, Fujian Medical University, Fuzhou 350004, China, ^2^ Department of Electronic Science, Fujian Provincial Key Laboratory of Plasma and Magnetic Resonance, Xiamen University, Xiamen 361005, China, ^3^ Laboratory of Radiobiology, Fujian Provincial Tumor Hospital, Fuzhou 350014, China, ^4^ Key Laboratory of Optoelectronic Science and Technology for Medicine, Ministry of Education, Fujian Normal University, Fuzhou 350007, China

Email: Yang Chen^*^- chzhy85@fjmu.edu.cn; Zhong Chen-chenz@xmu.edu.cn; Ying Su-zjsuying@hotmail.com; Donghong Lin-lindh65@163.com; Min Chen-cmjy503@163.com; Shangyuan Feng-syfeng@fjnu.edu.cn; Changyan Zou-312988066@qq.com

^*^ Corresponding author





**Figure S1. Individual 600 MHz ^1^H NMR spectra of cell extracts (A) and media (B).**





**Figure S2. Representative 600 MHz ^1^H NMR spectra of cell extracts (A~C) and cultured media (D~F).** **A** and **D** for nasopharyngeal normal NP69 cell, **B** and **E** for high differentiated CNE1 cell, **C** and **F** for low differentiated CNE2 cell.

**Table S1.** Assignments of metabolites from ^1^H NMR spectra of NPC cell extracts and cultured media

| **Metabolite** | **^1^H Shift (multiplicity)** | **Sample** |
| --- | --- | --- |
| acetate | 1.92(s^a^) | C^b^, M |
| acetone | 2.23(s) | M |
| adenine | 8.12(s), 8.13(s) | C, M |
| adenosine | 6.03(t) | C |
| ADP | 5.99(m), 7.83(s) | C |
| ATP | 5.62(m), 6.11(d), 8.24(s), 8.54(s), 8.58(s) | C |
| alanine | 1.49(d), 3.77(q) | C, M |
| choline | 3.20(s), 3.54(dd) | C |
| creatine | 3.04(s), 3.93(s) | C, M |
| ethanol | 1.18(t), 3.66(q) | C, M |
| formate | 8.46(s) | C, M |
| fumarate | 6.52(s) | C |
| glucose | 3.24(dd), 3.36(m), 3.42(m), 3.54(dd), 3.77(m), 3.84(m), 3.90(dd), 4.57(t) | C, M |
| glutamate | 2.06(m), 2.35(m), 3.76(dd) | C, M |
| glutamine | 2.14(m), 2.55(m), 3.78(t) | C, M |
| glycerol | 3.54(m), 3.66(m), 3.77(m) | C |
| GPC | 3.22(s), 3.60(m), 3.90(m), 4.29(m) | C |
| glycine | 3.56(s) | C, M |
| GTP | 5.52(m) | C |
| HEPES | 2.80(d), 2.83(m), 2.95(m), 3.03(m) | C |
| histidine | 3.16(dd), 3.98(dd), 7.07(s), 7.83(d) | C, M |
| 3-hydroxybutyrate | 1.25(s) | C |
| inosine | 8.19(s), 8.34(s) | C |
| *myo*-inositol | 3.28(t), 3.53(dd), 3.65(t), 4.06(t) | C, M |
| isobutyrate | 1.22(d) | C, M |
| isoleucine | 0.94(t), 1.02(d) | C, M |
| lactate | 1.33(d), 4.12(q) | C, M |
| lipids | 0.88(br), 6.83(br) | C |
| leucine | 0.96(t) | C, M |
| lysine | 1.41(m), 1.73(m), 1.83(m), 3.02(t) | C, M |
| maleate | 5.52(m) | C |
| methionine | 2.16(m), 2.65(t), 3.82(dd) | C, M |
| methylmalodate | 1.23(d) | M |
| phenylalanine | 3.98(dd), 7.33(d), 7.43(d) | C, M |
| phosphorylcholine | 3.23(s), 3.62(t) | C, M |
| proline | 2.02(m), 2.07(m), 2.36(m), 3.42(t) | C, M |
| pyruvate | 2.45(s) | C, M |
| serine | 3.82(dd), 3.90(m) | C |
| succinate | 2.40(s) | C, M |
| taurine | 3.28(t), 3.42(t) | C |
| threonine | 3.54(d), 4.23(m) | C, M |
| tryptophan | 7.13(m), 7.27(m), 7.54(d), 7.68(d) | C |
| tyrosine | 3.02(dd), 3.16(dd), 3.96(dd), 6.91(d), 7.20(d) | C, M |
| uridine | 5.91(m) | C |
| UTP | 5.52(m) | C |
| valine | 1.00(d), 1.05(d) | C, M |

^a^ Multiplicity: br: broad resonance; d: doublet; dd: doublet of doublets; m: multiplet; q: quartet; s: singlet; t: triplet.

^b^ C: cell extracts; M: media.

**Table S2.** Concentrations of characteristic metabolites derived from cultured media

| **Metabolite** | **NP69** | **CNE1** | **CNE2** |
| --- | --- | --- | --- |
| acetate | 3.07±0.23 ^†^ | 1.69±0.03 ^a^ | 0.22±0.03 ^a,b^ |
| acetone | 0.01±0.00 | 0.08±0.00 ^a^ | 0.07±0.00 ^a,b^ |
| adenine | 0.03±0.00 | 0.01±0.00 ^a^ | 0.01±0.00 ^a^ |
| α-glucose | 9.12±0.42 | 10.55±0.71 | 3.48±1.12 ^a,b^ |
| alanine | 0.33±0.04 | 0.45±0.01 ^a^ | 0.49±0.04 ^a^ |
| creatine | 1.94±0.20 | 0.04±0.00 ^a^ | 0.03±0.00 ^a,b^ |
| ethanol | 2.08±0.24 | 0.85±0.02 ^a^ | 0.76±0.04 ^a,b^ |
| formate | 0.02±0.00 | 0.01±0.00 ^a^ | 0.01±0.00 |
| glutamate | 2.31±0.08 | 3.29±0.06 ^a^ | 2.24±0.18 ^b^ |
| glutamine | 3.80±0.13 | 3.04±0.09 ^a^ | 1.69±0.19 ^a,b^ |
| glycine | 0.09±0.01 | 0.15±0.00 ^a^ | 0.11±0.01 ^b^ |
| histidine | 0.06±0.00 | 0.06±0.00 | 0.04±0.00 ^a,b^ |
| isoleucine | 5.02±0.30 | 5.01±0.09 | 3.10±0.17 ^a,b^ |
| lactate | 4.50±0.30 | 7.96±0.83 ^a^ | 14.08±0.88 ^a,b^ |
| leucine | 1.80±0.07 | 2.87±0.10 ^a^ | 1.46±0.24 ^b^ |
| lysine | 14.07±0.54 | 4.57±0.08 ^a^ | 2.43±0.16 ^a,b^ |
| methionine | 1.12±0.04 | 0.95±0.02 ^a^ | 0.55±0.06 ^a,b^ |
| methylmalodate | 6.37±0.26 | 0.34±0.01 ^a^ | 0.32±0.02 ^a^ |
| *myo*-inositol | 1.42±0.06 | 2.05±0.10 ^a^ | 0.75±0.21 ^a,b^ |
| phenylalanine | 0.04±0.00 | 0.29±0.01 ^a^ | 0.22±0.02 ^a,b^ |
| phosphocholine | 0.34±0.01 | 0.07±0.00 ^a^ | 0.05±0.00 ^a,b^ |
| proline | 2.41±0.08 | 3.93±0.09 ^a^ | 2.77±0.22 ^b^ |
| pyruvate | 0.08±0.01 | 0.16±0.01 ^a^ | 0.10±0.02 ^b^ |
| succinate | 0.71±0.02 | 0.12±0.00 ^a^ | 0.12±0.01 ^a^ |
| threonine | 0.05±0.00 | 0.25±0.01 ^a^ | 0.20±0.01 ^a^ |
| tyrosine | 0.02±0.00 | 0.15±0.00 ^a^ | 0.10±0.01 ^a^ |
| valine | 0.91±0.03 | 1.23±0.02 ^a^ | 0.95±0.07 |

^†^The concentrations of metabolites are presented as mean±SE of the integration value of the characteristic resonance of each metabolite.

Characteristic metabolite with *P*<0.05 versus: ^a^ normal NP69, ^b^ high differentiated CNE1.

**Table S3.** Main Raman band positions and the corresponding assignments of single living cells

| **Cell line** | | | **Assignments** | **Molecular origin** |
| --- | --- | --- | --- | --- |
| **NP69** | **CNE1** | **CNE2** |  |  |
| 408 | 408 | 408 | PI | lipids |
| 457 | 457 | 457 | phenyl (2) (Trp) | protein |
| 497 | 497 | 497 | S-S str. (Arg) | protein |
| 523 | 520 | 520 | S-S str., PS | protein, lipids |
| 621 | 620 | 620 | Phe | protein |
| 644 | 644 | 644 | C-C tw. (Phe, Tyr) | protein |
| 716 | 718 | 718 | A | DNA |
| 758 | 758 | 758 | Trp, T | protein, DNA |
| 782 | 781 | 781 | C/U ring br. | RNA |
| 827 | 830 | 830 | Out of plane ring br. (Tyr), O-P-O str. | protein, DNA |
| 852 | 852 | 852 | C-C ske. (Tyr) | protein |
| 935 | 939 | 939 | C-C str., protein backbone (α-helix conformation) (Pro, Val), glycogen | protein |
| 1003 | 1003 | 1003 | Phe | protein |
| 1032 | 1032 | 1032 | Phe | protein |
| 1041 | - | - | G | DNA |
| 1126 | 1126 | 1126 | GlcNac | protein |
| 1158 | 1157 | 1157 | C-C (& C-N) str. | protein, carotenoids |
| 1174 | 1173 | 1173 | Tyr | protein |
| 1207 | 1208 | 1208 | Trp, Phe, A, T | protein, DNA |
| 1317 | 1318 | 1318 | collagen, G | protein, DNA |
| 1337 | 1337 | 1337 | A, G, C-H def. | protein, DNA |
| 1448 | 1449 | 1449 | - | protein, lipids |
| 1605 | 1605 | 1605 | C=C in-plane bending mode (Phe, Tyr) | protein |
| 1656 | 1657 | 1657 | amide I | protein |

A: adenine; Arg: arginine; br.: breathing; def.: deformation; G: guanine; Phe: phenylalanine; PI: phosphatidylinositol; PS: phosphatidylserine; Pro: proline; ske.: skeletal; str.: stretching; T: thymine; Trp: tryptophan; tw.: twist; Tyr: tyrosine; Val: valine.
